# The Social Meanings of Artifacts: Face Masks in the COVID-19 Pandemic

**DOI:** 10.3389/fpubh.2022.829904

**Published:** 2022-04-14

**Authors:** Franziska Schönweitz, Johanna Eichinger, Janneke M. L. Kuiper, Fernandos Ongolly, Wanda Spahl, Barbara Prainsack, Bettina M. Zimmermann

**Affiliations:** ^1^Institute of History and Ethics in Medicine, School of Medicine, Technical University of Munich, Munich, Germany; ^2^Institute for Biomedical Ethics, University of Basel, Basel, Switzerland; ^3^Centre for Sociological Research, KU Leuven, Leuven, Belgium; ^4^Michael Smurfit Graduate Business School, University College Dublin, Dublin, Ireland; ^5^Centre for the Study of Contemporary Solidarity, Department of Political Science, University of Vienna, Vienna, Austria

**Keywords:** face mask, COVID- 19, pandemic, social meaning, artifact

## Abstract

Since the beginning of the COVID-19 pandemic, research has explored various aspects of face mask use. While most of the research explores their effectiveness to prevent the spread of the virus, a growing body of literature has found that using face masks also has social meaning. But what social meaning does it have, and how does this meaning express itself in people's practice? Based on 413 qualitative interviews with residents in five European countries (Austria, Belgium, Germany, Ireland, and Switzerland), we found that the meanings of face masks have changed drastically during the first months of the pandemic. While in spring 2020 people wearing them had to fear stigmatization, in autumn of 2020 not wearing masks was more likely to be stigmatized. Throughout the first year of the pandemic, we found that mask wearing had multiple and partly seemingly contradictory meanings for people. They were perceived as obstacles for non-verbal communication, but also a way to affirm friendships and maintain social contacts. They also signaled specific moral or political stances on the side of face mask wearers and non-wearers alike, expressed their belonging to certain communities, or articulated concern. In sum, our findings show how face masks serve as scripts for people to navigate their lives during the COVID-19 pandemic. We conclude that public and political discussions concerning face masks should include not only evidence on the epidemiological and infectiological effects of face masks, but also on their social meanings and their social effects.

## Introduction

By now, it has been well established that people's assessment of face mask effectiveness to protect them from infection is only one of a variety of factors that influence compliance with wearing mouth-and-nose coverings (“face masks”) (1–6). The wider social meanings of these face masks also play a significant role in compliance with mask mandates or recommendations (7).

These social meanings, in turn, are shaped by cultural and social norms, and by people's personal and social experiences with face masks. For example, people living in some world regions had already been wearing face masks routinely pre-COVID to avoid passing on germs in public spaces, while in other countries and regions face masks were associated almost exclusively with occupational health or hospitals. By analyzing 38 countries to identify global trends in face mask usage, Badillo-Goicoechea et al. (8) shed light on the diversity in face mask uptake. While mask usage was constantly high in several countries of Latin America and Asia, it remained low in Northern European countries such as Denmark, Sweden, or Norway (8). Badillo-Goicoechea et al. (8) identified strict masking policies in countries as one reason for higher mask usage, but also related behavioral factors like grocery shopping or going to a pharmacy with it, while behaviors associated with social occasions like going out for dinner, meeting friends outside one's home, going to work or for shopping with lower mask usage. They further found that older age, female gender, education and urbanicity were positively correlated with higher mask usage (8).

In this article, we provide evidence on the rather nuanced views and meanings that mask wearing had for people in five European countries during the first year of the COVID-19 pandemic. We draw upon a total of 413 qualitative interviews with residents in Austria, Belgium, Germany, Ireland, and Switzerland. We interviewed the same individuals twice, at two different time points: The first data collection period was in April 2020 when solid scientific evidence on the effectiveness and efficacy of face masks was still lacking, and the second in October 2020, when there was a general consensus in most of the scientific community that face masks could effectively contribute to pandemic containment (9–15).

In April 2020, when we carried out our first round of interviews (T1), the wearing of face masks was not mandatory but recommended in the participating countries. Only Austria had made face mask use mandatory in supermarkets, public transportation, and some other public buildings from 1 April. About a month later, on 29 April, mask mandates were introduced also in Germany. In Belgium, mask mandates were implemented in a limited form for public transport. Switzerland actively decided against making masks mandatory, but mask use was recommended as of 30 April 2020. At the time of our second round of interviews (T2), in October 2020, mask wearing was also mandatory in Ireland for public transport (10 July 2020) and retail (10 August). The extension of mask mandates for retail was also the case in Belgium (11 July). In Switzerland, compulsory mask wearing for public transport was introduced on 6 July but broader, nation-wide face mask mandates for public places were only implemented in October 2020.

## Literature Review

Besides a large number of studies analyzing the usefulness of face masks to counteract the COVID-19 pandemic (16–19), there is also a growing body of work investigating the social impact and influence of mask-wearing in the context of the COVID-19 pandemic. Some of the latter studies have explored factors influencing people's perceptions on face masks. Some, including data from Spain, the United States, the United Kingdom, Japan, Brazil, Denmark, Turkey, Canada etc., found differences in mask perceptions based on area, age, or gender: In most studies, mask wearing was associated with older age, female gender and living in urban areas (8, 20–23). Asri et al. (24) found that the motivations to wear a mask were changing with an increased age: “[w]hile for older people, mask wearing habits are best explained by their self-regarding risk preferences, younger people are also motivated by other-regarding concerns.” Similarly, a quantitative study carried out in Canada by Van der Linden and Savoie (25) found that their participants not only wore masks to protect themselves but also others.

Some studies found that higher individual COVID-19 risk perception was a motivating factor to wear face masks (20, 21, 26, 27). They indicate that risk perceptions significantly influenced the frequency of mask wearing in countries such as Spain, the United States and the United Kingdom (20, 21, 26, 27). In Spain, this was especially the case when fear increased due to disease related distress or the fear to infect a close relative or friend who had pre-existing health issues (20). In participants who were less concerned about infections, mask wearing decreased (20). Moreover, higher levels of formal education (8) and high level of trust in authorities (28) were found to predict mask-wearing.

Some studies found that mask-wearing was a form of social commitment, such as Betsch et al. (29) in Germany. Lu et al. (3) found the level of collectivistic thinking in in different US states to be a robust predictor for a high uptake of face masks. Where individualistic attitudes were dominant, mask use was lower (2, 3). This finding was also confirmed by Kemmelmeier and Jami (2) when it comes to the US. Powdthavee et al. (30) concluded that social identity together with in-group favoritism could be a primary reason for people to decide in favor or against mask wearing in the US, depending on the attitude of their social connections.

Face masks were also found to hinder social interaction. An Italian and a further international study analyzed the impact of mask wearing on interpreting facial expressions, which became especially difficult for men (31, 32). They concluded that females overall performed better since their ability to interpret facial expressions was more pronounced, which is also confirmed by previous studies without face masks (33). This lack in correctly interpreting facial expressions led to a social barrier and ultimately hinders social interaction.

Biermann et al. looked specifically at the effect of mask wearing on trustworthiness in Germany. They concluded that “a negative bias in trustworthiness appraisals of faces with a positive emotional expression covered by [mouth-nose-covers] is linked to a participant's evaluation of [mouth-nose-coverings] as inefficient and burdening and their experience of high psychological distress” (34). A study in Italy by Caniato et al. (35) investigated speech intelligibility in classrooms when students and teachers wore face masks, and found that masks overall negatively influence voice propagation especially when it comes to the voice range of male students and regardless of varying indoor acoustic characteristics. Further negative influences of wearing face masks and the correlation with social anxiety and mental health were identified by Saint and Moscovitch (36) in their exploratory review. A Taiwanese study by Chin et al. (37) shed light on the importance of fear as a driving factor for the public during the COVID-19 pandemic to adhere to protection measures.

Some studies looked at the influence of mass or social media coverage (38–40) and campaigning efforts: In their quantitative study Bokemper et al. (41) showed evidence from the US and Italy for communicating the effectiveness of face masks to protect others, which increased the usage of face masks more than communicating the protection benefits of the wearer. They also found that increased mask wearing promoted this behavior, acted as positive reinforcement and did not fuel free riding behavior (41). Kahane (42), in contrast, showed the impact of politicizing face masks in the light of the recent presidential election in the United States, where masks were used as a symbol for weakness by the former president Donald Trump and that mandatory mask use in individual states has nonetheless had a positive impact on overall mask use.

A number of studies looked specifically into cultural meanings of mask-wearing. Badillo-Goicoechea et al. global study on data from 2020 concluded that there must be some underlying cultural influences that does not map onto geographical regions: Some countries that had a consistently high face mask usage, such as Italy, Spain, Japan or Argentina. Others transitioned from a low to high usage rate like Portugal, Germany, Switzerland, Ireland or Canada. Third, they were those with a constantly low level like Denmark, Sweden and Norway, finally those with an irregular trend such as Russia, Australia, Austria or Poland (8). Casola et al. (43) emphasized the need to further analyze mask wearing behavior through a social-ecological lens which includes societal structure, community norms but also individual characteristics.

In sum, cultural and social norms around face masks have had significant effects in this pandemic. Especially in its early phases, mask wearers presumed to be from Asian descent faced aggression and violence from people who held “Asian looking” people responsible for importing or spreading the virus. Some refrained from wearing face masks out of fear that they, too, would become a target (44–47). Only when the wearing of face masks became mandatory for everyone, the face mask as an artifact lost its status of a “symbol of disease” and took on other meanings–for example, as symbols of prevention, or symbol of oppression (1). Still, the symbolic power of face masks remains.

Kabir et al. (48) referred to a “diverse and rich social dilemma structure” that influences face mask wearing and therefore highlighted the sociological dimensions of mask policies. Also, a article of Timpka and Nyce (49) referred to the significance of cultural as well as symbolic meaning, which should be considered by governmental decisions when passing regulations on mandatory mask wearing. Our own work aims to further contribute to a better understanding of the multiple meanings that are attached to, and shaping, face mask use.

## Materials and Methods

This publication is a result of the joint work of the members of the “Solidarity in times of a pandemic: What do people do, and why?” (SolPan) research commons. SolPan is a qualitative longitudinal and comparative research project across ten European countries (Austria, Belgium, France, Germany, Ireland, Italy, the Netherlands, Portugal, Switzerland, and the United Kingdom). Each country team conducted in-depth interviews with residents, lasting approximately 60 min on average. Participants were initially recruited through announcements on institutional websites and social media. To get a maximum variety of perspectives, we ensured demographic variability (age, gender, income, living area, living conditions, employment status, and education) by using purposive and snowball sampling at a later stage. The second round of interviews was a follow-up with the same participants. Here, some no longer took part. If we received feedback on why they no longer wanted to participate, it was mostly due to time or personal reasons. Accordingly, the numbers of participants were lower in the second round of interviews than in the first. In addition, participants moved. For example, one participant in the second round of interviews was no longer counted as a German-Swiss interviewee but as a German one. Interviews in both collection periods (in April 2020, T1, and October 2020, T2) were based on common interview guides across all countries (50) that aimed to capture people's practices and experiences during the ongoing pandemic as well as their motivations and attitudes toward the policy measures to contain the COVID-19 virus. Each country team translated the topic guide (one for each collection period) and asked questions on the background of the respective policy and societal context. The order of the open-ended questions followed the flow of the conversation. Interviews were conducted via telephone or video conference tools. SolPan received ethics clearance from the University of Vienna Ethics Committee (Ref.Nr.: 00544), the Technical University of Munich Ethics Committee (Ref.Nr.: 208/20 S), the KU Leuven Ethics Committee SMEC Approval (Ref.Nr.: G-2020 04 2007) and the University College Dublin Ethics Committee (Ref.Nr.: HS-E-20-70-Galasso). In terms of the Swiss interviews an ethics approval was not legally required [see Swiss Human Research Act (HRA)]. To facilitate data analysis across countries, verbatim transcripts were coded according to topics as identified within a common coding scheme (51), using the qualitative data analysis software Atlas.ti and NVivo. For a detailed description of the SolPan methodology see (52, 53).

As noted, this article builds upon data from 413 interviews in five countries: Austria (T1: 80, T2: 72), Belgium (T1: 36, T2: 23), Germany (T1: 46, T2: 43), Ireland (T1: 32, T2: 25), and German-speaking regions of Switzerland (T1: 31, T2: 25). The data allowed us to compare findings across countries and across time. While in April 2020 the use of face masks was a new practice to large parts of the European population, October 2020 marks a time of both habituation with the use and contestations revolving around the abolition and (re-)introduction of this policy measure.

This aricle's data analysis proceeded in four steps. First, we selected relevant passages from the interviews. While the interview guide for T1 did not specifically ask about face masks, many participants spontaneously referred to them. This was sometimes the case due to probing and follow-up questions. Most elaborations on face masks sparked from general questions about how people protected themselves and how they evaluated containment measures. For the T2 interviews, the SolPan interview guide and coding scheme was adapted to include a code for face masks. Participants were asked if they have worn a mask, and why (not), as well as inquiring into the purpose of wearing a mask. All passages referring to face masks in both T1 and T2 were chosen for more detailed analysis.

As a second step, all authors of this aricle sighted the selected interview data for their country in an inductive manner. We met to discuss the different impressions as well as emerging commonalities across countries.

Thirdly, based on these emerging commonalities and differences, the lead authors developed a memo guide that served as framework for the country specific analyses and ensured comparability (see Supplemental Material). The guide instructed all authors to analyze their country's data along two thematic blocks: (1) attributing meanings to face masks (attribution of effectiveness, symbolic meanings, moral meanings, social meanings) and (2) the relationship with recommendations and mandates. Authors were asked to add relevant citations from their interview passages for each analytical segment. Additionally, we asked all authors to reflect and write differences between T1 and T2 and compiled policy decisions concerning face masks in each country, drawing upon legal texts and policy research for data triangulation. The analyzing authors were invited to include other aspects relevant for their countries but not covered in the memo guide.

Fourth, accompanied by both senior authors, the first author summarized these country memos and reorganized them, identifying dominant themes and patterns across countries, and considering the differing face mask policies in the participating countries. These themes were subsequently discussed and refined by all authors. Finding that these themes all connect to socio-cultural meanings of face masks, we reorganized the findings in the themes presented below in the findings.

## Results

Based on our analysis and comparison of the qualitative interview data of residents from Austria, Belgium, Germany, Ireland, and German-speaking Switzerland, we now illustrate how the perceptions toward face mask wearing changed from April to October 2020, how face masks served as communicative tools that both fostered and hindered social relationships, and how face masks were seen as markers of the (ab)normal.

### A Fundamental Change in the Perception Toward Face Masks

#### Skepticism and Uncertainty in April 2020

How participants perceived the usefulness and necessity of face masks changed strongly from April to October 2020. In April 2020, many participants across all countries said that they were uncertain about the usefulness and effectiveness of face masks in protecting people from infection:

“No, I do not wear a mask. I wash [my hands] more often than I used to, of course.” (DE200414BZ03).

Even after public authorities officially recommended mask wearing in Spring 2020, some participants remained skeptical about their actual effectiveness and argued that keeping a physical distance to other people would be sufficient. Especially in Ireland and Switzerland, participants criticized the lack of clear communication from public authorities on whether face coverings were protective and should be worn:

“If you had said to me six months ago who would you trust on whether to wear a mask or not, I'd probably say the World Health Organization, but you know some of their information has been changing and not as clear cut as you would like, but again if you read the detail of it you can see that they do give you explanation.” (IE200427IG03).

In Germany, Belgium and Switzerland, participants also expressed the feeling of having been fooled, or lied to, by their government:

“(…) And now I have the feeling, they lied to us like you do to toddlers while it was so clear that they were lying. And then they say yes yes but we followed the guidelines of the WHO“, but yeah. A bit of common sense could have known that that face mask could really help a bit.” (T1_BE_JMLK03).

In some instances, participants also said they did not want to wear a mask as masks were in short supply and should be reserved for healthcare workers and others who needed them more urgently. In Belgium it was even forbidden to sell surgical masks to private parties (54).

#### Acceptance as a Protective Tool in October 2020

By October 2020, most participant had gotten used to wearing face masks and reported that they were wearing them on a regular basis. Some emphasized that they felt safer in public when wearing a mask:

“Now, of course, I feel more comfortable, especially when I'm out in public. But it almost has more to do with the other people. Because the other people don't pay attention [to the virus]. I have to look after myself and then I just put on the mask and then I feel safer. Yes.” (2CH201008BZ03).

Some participants emphasized that they would wear a mask not only to protect themselves but also out of concern for others. In fact, most of our participants who did wear masks early in the pandemic did so–not exclusively, but first and foremost–to protect others. The wearing of masks became a symbol of solidarity, which increased from April 2020 to October 2020.

“But then if we see people passing by we put it on, even if it doesn't help in open air or it has little use. But just out of respect for the other people by sayinglook, voila, we follow the rules and you don't have to be scared of us. (…) To show others like ok, we understand that certain people wish this or feel more safe if both parties wear a facemask. And that's the only reason for us.” (T2_BE_GM02).

“[…] so mask, I think that makes sense. It just has to be clear to everyone that you're protecting the other person and not yourself. If everyone does that, then everyone gets something out of it, so that's also logical.” (DE200501AS11).

Now that we have outlined how the perception towards face masks changed, we will take a closer look at the functions that masks had for people, and how they have scripted people's movements and interactions.

### Face Masks as Communicative Tools

In our data, participants reported that face masks influenced their social relationships and communication by both facilitating and hindering social interaction.

#### Communicating Non-verbally

First, face masks became an important tool for non-verbal communication. Particularly at the early stages of the pandemic, participants reported that mask-wearing was a signal that the wearer of a mask was aware of the pandemic situation and took it seriously. Another function of face mask wearing was to reminded others of the same. By October 2020, masks were still perceived as a constant reminder of the pandemic situation in Germany, Austria, and Switzerland, but unlike in April, participants more frequently referred to masks as creating some sense of safety and, therefore, a form of “psychological protection” (2DE201006NH04). Several participants said that masks enabled people to maintain social contacts and go to places where they would otherwise not feel safe to go:

“In this respect, that is perhaps a rather egoistic reason, if you like. It's not just to protect the other person, but I think I / If I can put it that way: I wear the mask to save a social situation that I otherwise would not have or would not enter into in that form, where I would otherwise say “Nah, then I don't want to meet.” Or then I don't go into a store now or wouldn't go into a restaurant now where I felt like the waiters are all not wearing a mask now. Then I think / I think that's socially problematic and I think I care a lot that we kind of maintain certain social contexts as long as we can. And I would like to contribute to making that possible.” (2DE201009NH11).

#### Demonstrating Moral Action

Second, face masks were a symbol for acting morally. While in April 2020 it was considered immoral to wear a face mask because they were scarce and their effectiveness unclear, many participants told us that they felt a kind of moral duty to wear a mask in October 2020. Wearing a face mask was now seen as an effective measure to protect others and oneself, especially in Belgium, Germany, and Switzerland.

“Well, if you believe the statements in the press and the virologists, then of course primarily to protect others from infection. And if everyone wears one, then of course you are also protected yourself. That's clear.” (2DE201012NH15).

Belgium participants further considered the wearing of a mask as polite and respectful, underscoring its nature as a considerate and solidaristic practice:

“Yes, just now someone stopped by to drop something off and he had that on. I then also put mine on. That I find just, really weird in relation to the elementary politeness. Such a new thing that we have learned of he has that on, I will then also quickly put that on. Not that I saw any reason.” (T2_BE_SV04).

For most of our participants, the moral economy of masks reversed from April to October 2020: While some considered it immoral wearing a mask in April, it was now immoral not to wear one.

#### Re-affirming Social Relationships

Third, face masks served to affirm social relationships. Particularly in April 2020 when they were scarce, some participants from Belgium, Germany and Austria spoke about people who made face masks at home for others. Some of our participants also participated in such initiatives themselves:

“But I already made a lot of face masks. Now I haven't done that the last two weeks. But it was for the husband of a colleague who is a cop, and they didn't have enough. So, I made face masks for them.” (T1_BE_EL02).

Some Austrian participants reported making particularly stylish or beautiful face masks to gift them to friends. Other participants from Belgium and Germany made masks for others arguing that they were simply needed. In this sense, face masks became a token of friendship or support, and even a means of communication: Making a face mask for someone said more than people could express in words. They served to reaffirm friendships and other relationships and signaled how important they were also (or perhaps especially) in times of crisis.

### Face Masks as Obstacles to Relations and Communication

#### Feeling and Being Alienated in April 2020

Face masks were not only seen as conducive to communication and prosocial activities, but they were also experienced as doing the opposite in some contexts. In April 2020, almost a quarter of our participants (except from Belgium participants) feared to be alienated from their social circles when wearing a mask when no one else did. This perception was particularly strong in Germany and Switzerland. Some also feared to be judged as egoistic when wearing a mask during a shortage in supply or being ridiculed for being overcautious:

“And how I protect myself was then just to go out with the mask, in the beginning, where I thought: I am an alien, here, in Switzerland.” People think: “Oh come on, we are in Switzerland and we have clean air here and nothing can happen to us.” (CH200428BZ20).

For example, one Swiss participant found that wearing a mask in the car was “hysterical.” By October, many saw not masking as a cause for stigma. Interestingly, there was a perceived postponement of stigma in Switzerland as masks were still not mandatory in October 2020 and as Swiss participants put it: if you voluntarily use one, people look at you as if you were sick or as if you felt morally superior.

This delayed attitude towards masks might be related to the late introduction of mandatory masking at the end of October in Switzerland, while countries that showed this stigma mainly in April 2020 introduced the masking obligations already in spring or summer.

#### Demonstrating Polarized Stances in October 2020

Another way in which masks were an obstacle for communication and friendly interactions was that they became a marker for a particular stance in the COVID-19 debate. Mask wearers were seen (and often also saw themselves) as demonstrating that they were taking the dangers of the pandemic seriously. By contrast, those who did not wear masks in places where it was recommended or mandatory to wear one were seen as making small, or even denying, the risks posed by COVID-19. Many participants voiced frustration and anger about the behavior of the other group (which in our sample were mostly those not wearing masks, because most of our participants did wear them).

“I have to say, to be honest, I'm angry at my fellow citizens, because the way some of them behave, I mean, most of them are well-behaved anyway, but the way some of them behave in the subway or in the supermarket, because of this stupid mask, that gets me so worked up. It makes me so angry when I see people who have the mask over their mouth but not over their nose. Because I think to myself, either stand by the fact that you don't want to wear the mask. Then remove it completely and at least be brave.” (2AT201012KP03).

In many instances, participants said they were too afraid to confront those not wearing a mask when they should. Women were concerned about verbal or even physical violence by (mostly male) mask refusers.

“You hear and read reports of people being assaulted because they remind others of the mask mandate. I think I would not have the courage to do that. Especially with young men, I think I would rather not tell them to put that piece of clothing into their face.” (2AT201013BP06).

“What I noticed, maybe in this context: When I see someone who doesn't have the mask on correctly, I don't dare to point it out to them. /I: And why?/ Because I don't want to provoke a conflict. […] Yes, then I take two steps back or give the person a wide berth and, yes, it does bother me. But unfortunately, these are then also, yes, people / Let's say so, to build up prejudices: They are often male.” (2DE201009NH09).

In the few instances, participants said that they did confront others about their mask behavior, communication was reported as ineffective and full of tension.

“P: There are some who vehemently refuse the mask and risk being thrown out of the supermarket. I: And did you also start a conversation with these people? P: Yes, of course, but it's extremely interesting because you can't say exactly why that is. So, they can't say it exactly themselves. The argument that it restricts personal freedom comes up immediately, and that is somehow the common thread that runs through the refusal.” (2AT201023CH09).

In some cases, however, people had merely forgotten to put the mask on. This complicates the dominant idea of two binary “camps” in public discourse: the fervent mask supporters and die-hard mask refusers.

#### Hindering Non-verbal Communication

Another way in which masks were seen as an obstacle to communication was very physical: They made it harder to see and read people's faces. Many Austrian, German and Swiss participants saw this “loss” of facial expressions as a loss of interpersonal relationships, fearing a growing distance towards others due to face masks.

“It's so strange. You can't really look people in the face anymore. You can't see their facial expressions. It always looks like danger, it's not good either, it signals that it's just not a normal state. And yes. Masks, that's good now. We should do that now, but I hope that it will no longer be necessary […].I have always found it very strange when Asians walk around with masks on and I think I would continue to feel that way.” (2CH201005JE01).

One Austrian participant told us how wearing a mask at a cultural event was hindering her from experiencing the freedom she usually does.

“And we danced, with masks. And it made me infinitely sad because I don't see the facial expressions. And that is non-verbal communication. And that is totally missing through these masks. So, I had to leave in between because it wasn't psychologically, I just couldn't stand it. And for me, culture, theater, music, opera is the greatest freedom. For me, it's freedom of the soul. There is no limit for me. And when I sit there with the mask, go there with the mask, sit there with the mask and I have the feeling that something is not right […].” (2AT201014SE05).

Some participants stated that masks were particularly difficult for people with mental impairment, the elderly, and children. In some instances, this could also be an obstacle to communication with members of these groups.

“Yes, and people with dementia or mental illness in particular sometimes don't understand why we come along masked in that manner. Yes, that is a real challenge.” (CH200420BZ02).

### Face Masks as Markers of the (Ab)normal

In April 2020, many participants referred to a sense of unease about the wearing of face masks. Some perceived them as foreign to European culture and feared they might have detrimental effects on social cohesion. In Austria, Germany, Belgium and Switzerland, there were participants spontaneously referring to Asian countries when discussing face mask wearing. Most perceived the regular use of face masks “in China,” or “in Asian countries,” as something they did not want to happen in their own culture.

“I fear that the distance between people could become greater. In China, or in the Asian countries, it has been increasing for years. In Japan, in metropolitan areas, even in Tokyo or in the larger cities in China, it's completely normal for people to go out onto the streets wearing masks.” (DE200417BZ07).

While triggering a sense of unease, oppression, and stigma, face masks, on the other hand, became a symbol for governmental trustworthiness and reliability, or at least for individual's relation towards the government. Particularly in April 2020, participant's willingness to wear masks and to accept the related discomfort was related to risk perception as well as their general trust in the government and public institutions, entities that primarily promoted public education regarding the wearing of masks. Those who had such trust or at least trusted the explanations given were more willing to wear masks-even if they found them very uncomfortable or were not fully convinced of their effectiveness.

By October 2020, most participants had accepted masks as part of the “new normal.” Masks became an artifact of everyday life and were now being perceived to be relatively easy to integrate into daily routines.

“It's also crazy how quickly you get used to things too. That's still impressive, with masks or other measures. That's suddenly so normal and then you think, yeah, are we maybe always going to walk around with masks?” (2CH201007BZ02).

“Yes in March, April, everything was brand new, and we all didn't know what was going on and how and what, but in the meantime for me it's now part of everyday life, now and then I forget to take the mask with me when I go shopping, but otherwise yes, it's just part of it now.” (2AT201014WS02).

Masks mandates facilitated the acceptance of face masks as normal. While some Swiss participants still complained about issues concerning social alienation and impaired communication due to face masks in October 2020, none of the participants in other countries (where broad national face mask mandates had already been in place) mentioned these issues anymore.

“If we had to [wear face masks] over several years, that would certainly be really bad. Because there is no mimic […] Before you could read in someone's face, how is he, what does he mean, what does he really think? All of this is missing.” (2CH201006JE02).

However, some of the Belgian participants considered the issuing of mandatory hygiene measures of any kind an expression of a lack of trust of the state in their citizens. German and Belgium participants felt that measures were “over-bureaucratized” instead of relying on the people and their natural ability to act in the right manner. One German participant expressed that she no longer wanted to be patronized by the government:

“I just find it, yes, completely unpleasant. If someone dictates to me where I may travel, what I may and may not do, and if an authority dictates to me that I must refrain from certain things that are not justified from my point of view / I can speak quite openly.” (2DE201016BZ05).

Still, upon broad mandates, participants got used to face masks and accepted them in their “new normal everyday life,” which is fundamentally different from the normality they knew before the pandemic.

## Discussion

We presented findings from our qualitative interviews with residents from five European countries, conducted in April and October 2020. While findings were similar among all participating countries, we noticed important individual differences within each country in terms of how people used and perceived masks. These differing views and perceptions, however, share the commonality that face masks served as scripts to navigate the pandemic.

In April 2020, mask-wearing attracted social attention as it was uncommon and signaled weakness or risk of contagion. Mask-wearers were perceived to be very sensible or even “hysterical,” according to one Swiss participant. A few participants referred to gender-related differences, with men being less likely to adopt face masks early, which is in line with previous quantitative studie's findings regarding gender differences in mask uptake. According to Haischer et al. (21). US American men perceived wearing face masks as an expression of weakness and shameful behavior. By contrast, women were reported to wear masks more frequently (21). While men in the Western world tended to perceive face masks as a limitation of their independence women mainly perceived masks as being uncomfortable (22). In this sense and in line with our findings, masks use conveyed personal and social values and while contributed as socially hindering factor they simultaneously functioned as a script to navigate the pandemic. They visibly divided the public not only from an ideological perspective, but also due to different risk perceptions, creating age- or gender-based camps.

In line with the studies mentioned (20, 21, 26, 27) in the literature review on the influence of risk our finding indicate that the usage of face masks served as a script that illustrated individual risk perception and compliance with governmental containment policies.

At the beginning of October 2020, the shortage in face mask supply was over and masks were mandated in public places in most participating countries (except for Switzerland). Consequently, masks became an artifact to use in daily life. The stigmatization of face mask wearers that marked beginning of the pandemic was no longer present, except for Swiss participants. Betsch et al. (29) confirmed our findings in showing that in Germany under voluntary circumstances masks were afflicted more by stigma than when they were mandatory. Our Participants explained that masks helped them to “save social interaction” as they enabled them to navigate back to some form of social normality. Signs and regularly updated information on where and when to wear face masks became an integrated aspect of the “new normal.” Furthermore, participants emphasized to wear masks not only for their own safety, but also to protect others, which confirms the findings of the studies mentioned in the literature review.

Concerning social relationships and communication, however, we found masks to fulfill multiple, and partly contradictory, functions. Their presence not only helped to maintain social contacts and signaled solidarity, but it also served to reaffirm social relationships, and created a sense of security, which increased the psychological wellbeing of participants. This notion is supported by Szczesniak et al. (55) who found that the perceived self-protection and solidaristic behavior in terms of face masks positively contribute to overall psychological wellbeing in Poland. Seen in this light, masks functioned not only as interpersonal navigational aids, but also as psychological ones. Yet, our findings further indicate that face masks, even though they became a daily artifact, remained an obstacle for social interactions and fostered a social divide. Accordingly, while face masks were perceived as a means to regain some freedom (e.g., to meet other people, go to the theater, etc.) they overall still remained obstacles to “normal” life as before the pandemic.

With increasing evidence on their effectiveness and the implementation of face mask mandates, the wearing of face masks became a social and moral duty for many of our participants. In line with this, Betsch et al. (29) found that the majority of the overall 925 German respondents perceived mask-wearing as prosocial behavior under voluntary as well as under mandatory circumstances. However, our study complicates these findings, showing that mask-wearing was not always perceived as prosocial, but indeed could also impair social relations and communication depending on the circumstances. For instance, some of our participants saw them as a barrier to especially non-verbal communication e.g., when it comes to the difficulty of correctly interpreting facial expressions. Supporting this finding, the two quantitative survey studies performed by Calbi et al. (31) and Freud et al. (32) state the difficulty in interpreting face expressions correctly while wearing a face mask, which ultimately leads to a potential social barrier.

In the Spanish national survey study from April 2020, Barceló and Sheen (20) found that participants were more likely to wear face masks in “[…] a region or province where mask wearing behavior is more common” (p.12). They explained strong effect of mask wearing regions on their habitants by a “neighborhood effect,”^1^ which influenced the sense of community and shaping social identity as well as political orientations. Our findings expand on this notion by showing how face masks served as non-verbal scripts that facilitated or hindered social exchange. Supported by the literature discussed in the beginning collective use of face masks seems to foster mask-wearing (2, 3).

Even though it was visible in our data that masks were widely adopted by people across all countries under study, it is questionable whether at some point we will fully accept that these circumstances in which we currently live may persist to some degree. If so, this would indeed lead to a turn for masks from being a marker for the “abnormal” towards a marker for “normalcy.” After all, masks became reminders of the constant presence of the pandemic situation (4, 58, 59).

## Limitations

This qualitative analysis does not claim quantitative generalizability. A combination of purposive and snowball sampling was used in the SolPan research commons to recruit participants from a wide range of demographics for an interview. Despite our best efforts, middle class, and more highly educated people were slightly overrepresented in our sample. No observational data was collected, but the findings derived from the analysis of the experiences the participants shared with us. Since the study was done online or via telephone due to COVID-19, results might have been influenced differently compared to face-to-face interviews. Through the SolPan research commons, we had the opportunity to compare several European countries, including Austria, Belgium, Germany, Ireland, and German-speaking Switzerland. Due to the nature of our research, we can offer plausible explanations for visible differences occurring between the countries, but cannot prove causal effects.

## Conclusion

With this qualitative inquiry about the change in the meaning and perception of face masks in the first months of the COVID-19 pandemic, our findings add nuance to the numerous existing quantitative inquiries on the motives of mask-wearing and meaning of face mask policies. While scientific proof on the effectiveness of communal mask-wearing for the containment of SARS-CoV-2 has become generally accepted by October 2020, their social and cultural meaning in Europe is significantly influencing their perception by the general population. We showed how face masks served as scripts to navigate the pandemic and how these scripts changed for the vast majority from being an alienated sign of danger to being a sign for security and freedom and as an enabler of social interaction during the pandemic.

A lesson to draw from our findings is the need for a broader understanding of “scientific evidence” regarding face masks. So far, scientific evidence is mostly limited to epidemiological and infectiological data. The systematic and scientific exploration of social and personal meanings is, however, a key part in shaping people's practices. Policy makers devising rules and measures including face mask use are well advised to learn about the different and nuanced ways in which face mask use has meaning for people, rather than thinking about compliance with mask mandates in a binary manner.

## Data Availability Statement

The raw data cannot be made available as interview transcripts contain personal information that cannot be fully anonymised.

## Ethics Statement

The studies involving human participants were reviewed and approved by University of Vienna Ethics Committee (Ref. no. 00544), Technical University of Munich Ethics Committee (Ref. no. 208/20 S), KU Leuven Ethics Committee SMEC Approval (Ref. no. G-2020 04 2007), University College Dublin Ethics Committee (Ref. no. HS-E-20-70-Galasso). The patients/participants provided their written informed consent to participate in this study. Written informed consent was obtained from the individual(s) for the publication of any data included in this article.

## Author Contributions

FS, JE, JK, FO, BP, WS, and BZ: initial data analysis, manuscript revisions of first draft, and final approval of submitted manuscript. FS: summary of country analyses, manuscript first draft, and coordination and organization of contributions. BP and BZ: supervision. All authors contributed to the article and approved the submitted version.

## Funding

This work was supported by the KU Leuven BOF SolPan Grant under Grant (3H200158); the Global Health Research in the Wake of the Sars-CoV-2 Outbreak Grant from the Federal Ministry of Education and Research in Germany under Grant (01KI20510); the University of Basel research grant (3BE1003) and the European Research Council (ERC) under the European Union's Horizon 2020 research and innovation programme (Grant No. 771217).

## Conflict of Interest

The authors declare that the research was conducted in the absence of any commercial or financial relationships that could be construed as a potential conflict of interest.

## Publisher's Note

All claims expressed in this article are solely those of the authors and do not necessarily represent those of their affiliated organizations, or those of the publisher, the editors and the reviewers. Any product that may be evaluated in this article, or claim that may be made by its manufacturer, is not guaranteed or endorsed by the publisher.

## Footnotes

^1^Neighborhood effect means a certain (geographic) proximity to a social network that ultimately influences the behavior of individuals (56). It can be seen as a “virtuous social conduct” whereas the question remains to what extend social conditions have to exist for such a local establishment (57).
