# Supplementary material for: The Social Meanings of Artifacts: Face Masks in the COVID-19 Pandemic
Source: Front Public Health. 2022 Apr 14;10:829904. doi: 10.3389/fpubh.2022.829904 (PMC9049272; doi:10.3389/fpubh.2022.829904)
Supplement: Supplementary file 1 [file Table_1.DOCX]

Supplementary Material

# Memo Guide for Country Data Analysis

Country: XX , Researcher(s): XX

**Deadline:** 31 May 2021

## Main Research Question:

## How did perceptions towards face masks change from April to October (beginning of November) 2020 and how was this related to country-specific policies about face masks?

## Tasks

*🡪 Please answer the questions for T1* ***(part 1)*** *and T2* ***(part 2)*** *by writing a short paragraph (embed data into country-specific policy landscape, if possible) and add fitting quotes*
*🡪 compare T1 to T2* ***(part 3)***

*****If you cannot make any statement on a question, feel free to leave it out***

## Policy Measures

Please write down a timeline including the most important policy decisions concerning face masks in your country (from the beginning of the pandemic until the beginning of November 2020)

| **Date / time frame** | **Policy measure** | **Source (e.g. official website)** |
| --- | --- | --- |
|  |  |  |
|  |  |  |
|  |  |  |
|  |  |  |

## Part 1: Analysis of T1

### 1. Attributing meanings to face masks

**1.1. What moral and emotional meaning did the wearing of face masks have for the participants?** *(e.g. face mask wearing as a solidaristic act / the least one can do / moral duty / assigning stigma)*

| *Paragraph…* |
| --- |

| **Quote** | **Reference Number** |
| --- | --- |
| *e.g. “Yes, I don't wear a mask or anything like that now, because I think that's enough with the distance. But I also understand it somehow when others wear them. Yes, exactly and just disinfect a bit and touch as little as possible outside”* | *e.g. DE200418BZ08* |
|  |  |
|  |  |

**1.2. What (symbolic) meanings did participants attach to the wearing of face masks?** *(e.g. political statement, “silent protest”)*

|  |
| --- |

| **Quote** | **Reference Number** |
| --- | --- |
|  |  |
|  |  |
|  |  |

**1.3. Was the wearing of face mask a marker for identification with a group? And if so, how did the wearing or not wearing of face masks changed their relationship to others?**

|  |
| --- |

| **Quote** | **Reference Number** |
| --- | --- |
|  |  |
|  |  |
|  |  |

### 2. Recommendations and mandates

**2.1. To what extend did the practice of wearing face masks go beyond (a) recommendations (b) mandates by public authorities?**

**(a)**

|  |
| --- |

| **Quote** | **Reference Number** |
| --- | --- |
|  |  |
|  |  |
|  |  |

**(b)**

|  |
| --- |

| **Quote** | **Reference Number** |
| --- | --- |
|  |  |
|  |  |
|  |  |

**2.2. To what extend did they remain below what public authorities (a) recommended (b) mandated?**

**(a)**

|  |
| --- |

| **Quote** | **Reference Number** |
| --- | --- |
|  |  |
|  |  |
|  |  |

**(b)**

|  |
| --- |

| **Quote** | **Reference Number** |
| --- | --- |
|  |  |
|  |  |
|  |  |

**2.3. To what extent was the willingness to wear face masks (based on recommendations) related to participants risk perception?** *(e.g. personal risk of getting infected / severely sick; societal risk of overburdened health care system, risk of close ones belonging to a risk group, etc.)*

|  |
| --- |

| **Quote** | **Reference Number** |
| --- | --- |
|  |  |
|  |  |
|  |  |

**2.4. In what instances did participants refer to instructions and communication of a) authorities and b) experts when referring to face masks?**

**(a)**

|  |
| --- |

| **Quote** | **Reference Number** |
| --- | --- |
|  |  |
|  |  |
|  |  |

**(b)**

|  |
| --- |

| **Quote** | **Reference Number** |
| --- | --- |
|  |  |
|  |  |
|  |  |

3. Other aspects *(Please share with us any other striking points that you noticed in T1 or when comparing answers to country-specific policies)*

|  |
| --- |

| **Quote** | **Reference Number** |
| --- | --- |
|  |  |
|  |  |
|  |  |

## Part 2: Analysis of T2

### 1. Attributing meanings to face masks

**1.1. What moral and emotional meaning did the wearing of face masks have for the participants?** *(e.g. face mask wearing as a solidaristic act / the least one can do / moral duty / assigning stigma)*

|  |
| --- |

| **Quote** | **Reference Number** |
| --- | --- |
|  |  |
|  |  |
|  |  |

**1.2. What (symbolic) meanings did participants attach to the wearing of face masks?** *(e.g. political statement, “silent protest”)*

|  |
| --- |

| **Quote** | **Reference Number** |
| --- | --- |
|  |  |
|  |  |
|  |  |

**1.3. Was the wearing of face mask a marker for identification with a group? And if so, how did the wearing or not wearing of face masks changed their relationship to others?**

|  |
| --- |

| **Quote** | **Reference Number** |
| --- | --- |
|  |  |
|  |  |
|  |  |

### 2. Recommendations and mandates

**2.1. To what extend did the practice of wearing face masks go beyond (a) recommendations (b) mandates by public authorities?**

**(a)**

|  |
| --- |

| **Quote** | **Reference Number** |
| --- | --- |
|  |  |
|  |  |
|  |  |

**(b)**

|  |
| --- |

| **Quote** | **Reference Number** |
| --- | --- |
|  |  |
|  |  |
|  |  |

**2.2. To what extend did they remain below what public authorities (a) recommended (b) mandated?**

**(a)**

|  |
| --- |

| **Quote** | **Reference Number** |
| --- | --- |
|  |  |
|  |  |
|  |  |

**(b)**

|  |
| --- |

| **Quote** | **Reference Number** |
| --- | --- |
|  |  |
|  |  |
|  |  |

**2.3. To what extent was the willingness to wear face masks (based on recommendations) related to participants risk perception** *(e.g. personal risk of getting infected / severely sick; societal risk of overburdened health care system, risk of close ones belonging to a risk group, etc.)?*

|  |
| --- |

| **Quote** | **Reference Number** |
| --- | --- |
|  |  |
|  |  |
|  |  |

**2.4. In what instances did participants refer to instructions and communication of a) authorities and b) experts when referring to face masks?**

**(a)**

|  |
| --- |

| **Quote** | **Reference Number** |
| --- | --- |
|  |  |
|  |  |
|  |  |

**(b)**

|  |
| --- |

| **Quote** | **Reference Number** |
| --- | --- |
|  |  |
|  |  |
|  |  |

3. Other aspects *(Please share with us any other striking points that you noticed in T2 or when comparing answers to country-specific policies)*

|  |
| --- |

| **Quote** | **Reference Number** |
| --- | --- |
|  |  |
|  |  |
|  |  |

## Part 3: Comparison of T1 and T2

**** There is no need to add further quotes for the comparison – only if you have anything to add**

### 1. Attributing meanings to face masks

**1.1. What moral and emotional meaning did the wearing of face masks have for the participants?** *(e.g. face mask wearing as a solidaristic act / the least one can do / moral duty / assigning stigma)*

|  |
| --- |

| **Quote** | **Reference Number** |
| --- | --- |
|  |  |
|  |  |
|  |  |

**1.2. What (symbolic) meanings did participants attach to the wearing of face masks?** *(e.g. political statement, “silent protest”)*

|  |
| --- |

| **Quote** | **Reference Number** |
| --- | --- |
|  |  |
|  |  |
|  |  |

**1.3. Was the wearing of face mask a marker for identification with a group? And if so, how did the wearing or not wearing of face masks changed their relationship to others?**

|  |
| --- |

| **Quote** | **Reference Number** |
| --- | --- |
|  |  |
|  |  |
|  |  |

### 2. Recommendations and mandates

**2.1. To what extend did the practice of wearing face masks go beyond (a) recommendations (b) mandates by public authorities?**

**(a)**

|  |
| --- |

| **Quote** | **Reference Number** |
| --- | --- |
|  |  |
|  |  |
|  |  |

**(b)**

|  |
| --- |

| **Quote** | **Reference Number** |
| --- | --- |
|  |  |
|  |  |
|  |  |

**2.2. To what extend did they remain below what public authorities (a) recommended (b) mandated?**

**(a)**

|  |
| --- |

| **Quote** | **Reference Number** |
| --- | --- |
|  |  |
|  |  |
|  |  |

**(b)**

|  |
| --- |

| **Quote** | **Reference Number** |
| --- | --- |
|  |  |
|  |  |
|  |  |

**2.3. To what extent was the willingness to wear face masks (based on recommendations) related to participants risk perception?** *(e.g. personal risk of getting infected / severely sick; societal risk of overburdened health care system, risk of close ones belonging to a risk group, etc.)*

|  |
| --- |

| **Quote** | **Reference Number** |
| --- | --- |
|  |  |
|  |  |
|  |  |

**2.4. In what instances did participants refer to instructions and communication of a) authorities and b) experts when referring to face masks?**

**(a)**

|  |
| --- |

| **Quote** | **Reference Number** |
| --- | --- |
|  |  |
|  |  |
|  |  |

**(b)**

|  |
| --- |

| **Quote** | **Reference Number** |
| --- | --- |
|  |  |
|  |  |
|  |  |

3. Other aspects *(please state any other differences that stroke you when comparing T1 to T2 or when comparing answers to country-specific policies)*

|  |
| --- |

| **Quote** | **Reference Number** |
| --- | --- |
|  |  |
|  |  |
|  |  |
